# Keyhole mitral valve repair: avoidance learning curve by using low-fidelity simulator

**DOI:** 10.1186/1749-8090-8-S1-P163

**Published:** 2013-09-11

**Authors:** A Hossien, I Khan, H Subhani, S Ashraf

**Affiliations:** 1 Cardiothoracic Surgery Department, Morriston Hospital, Swansea, UK

## Background

There are several benefits of performing the mitral valve (MV) repair in minimally invasive technique, but it is considered difficult due to various reasons e.g. concerns of inferior outcome results compared to conventional technique, concerns of impaired patient safety and poor training. Therefore we propose a portable simulator for cardiac surgeons to be proficient in MV surgical techniques and to minimize the learning curve.

## Methods

The minimal invasive MV simulator was made from a MV model that was placed inside a box. The MV is self-constructed model was tested to simulate the flexible property of the normal MV components. The rings and valves were made from available materials, which have been used effectively in MV repair and replacement. The building process is detailed in this study. Total cost was calculated in Euros. The number of procedures performed on the same model was recorded.

## Results

Repetitive practising using the MV simulator leads to establish basic techniques in minimally invasive environment e.g. handling of the instruments, endoscopic knotting with needle, endoscopic knotting with knot-pusher, tissue handling and dissection and Placements of stitches on the annulus. The usage of the simulator results in performing of all surgical procedures including MV ring annuloplasty, Quadrangular and triangular resection, Edge to Edge, neo artificial chordae, pericardial augmentation of the leaflets and MV replacement as well. Unrestricted number of procedures was performed after covering the sponge with surgical tape to enhance effective manipulation.

## Conclusions

The surgical skills in minimally invasive MV surgery can be established by usage of low fidelity simulator. The familiarity in performing surgical procedures results in reducing time consumed in the operation room and reduction of the learning curve. The high cost of the training in keyhole surgery can be reduced effectively through the use of this low cost simulator.

